# Pressure-Regulated Volume Control and Pressure-Control Ventilation Modes in Pediatric Acute Respiratory Failure

**DOI:** 10.5152/TJAR.2021.1412

**Published:** 2022-02-01

**Authors:** Hasan Serdar Kıhtır, Nihal Akçay, Esra Şevketoğlu

**Affiliations:** 1 Department of Pediatric Critical Care, University of Health Sciences Antalya Training and Research Hospital, Antalya, Turkey; 2 Department of Pediatric Critical Care, University of Health Sciences Bakırköy Dr. Sadi Konuk Training and Research Hospital, İstanbul, Turkey

**Keywords:** Artificial respiration, pediatric intensive care units, positive-pressure respiration, respiratory insufficiency

## Abstract

**Objective:**

The objective of this study is to present our experience using the pressure-regulated volume control and the pressure-control ventilation modes in children.

**Methods:**

Patients with acute respiratory failure ventilated with pressure-regulated volume control or pressure-control modes were retrospectively evaluated. The patient’s ventilation parameters (of the first 7 days of ventilation or of the whole ventilation period, if the patient had been ventilated less than 7 days), SpO_2_, blood gases, and demographic data were collected from the pediatric intensive care unit database.

**Results:**

Sixty-one patients (median age 12 [4.8-36.4] months) were enrolled in the study. The pressure-control ventilation mode was used on 40 patients (65.6%) and the pressure-regulated volume-control mode was used on 21 (34.4%) patients. Twenty-eight patients (45.9%) had hypoxemic respiratory failure and 44 (72.1%) had hypercapnic respiratory failure. The median positive end-expiratory pressure was higher in pressure-control ventilation mode (5.4 [4.2-6.3] cmH_2_O) than the pressure-regulated volume-control mode (4.05 [3.68-4.41] H_2_O, *P* < .001). Pressure-control mode was used more frequently in hypoxemic cases but both modes were used equally in hypercapnic cases. Hypoxic respiratory failure (yes/no), odds ratio: 3.9 (95% CI 1.2-12.3, *P* = .02), Ph (nadir), odds ratio: 0.004 (95% CI 0.000-0.275, *P* = .01), and base excess, odds ratio: 0.88 (95% CI 0.79-0.98, *P* = .02) were associated with intensive care mortality.

**Conclusions:**

Although the pressure-control ventilation mode was preferred more frequently in hypoxemic respiratory failure, there was no significant difference between the 2 respiratory modes in terms of length of pediatric intensive care unit stay, MV duration, and mortality. The pressure-regulated volume-control mode seems to be a safer option for physicians who do not have enough experience in using pressure-control ventilation mode.

**Main points**

1. Pressure-regulated volume-control mode provides volume-targeted ventilation with a pressure-controlled breathing pattern and its popularity is increasing in Turkish pediatric critical care units.
2. It is not clear whether the pressure-regulated volume-control mode is superior to the pressure-control mode.
3. In our study, children ventilated with pressure-regulated volume-control mode had similar outcomes with patients ventilated in pressure-control mode.
4. Pressure-regulated volume-control mode appears to be a safe option for pediatric patients, especially in inexperienced centers.

## Introduction

Pediatric Mechanical Ventilation Consensus Conference does not have a specific ventilation mode proffer in children with acute respiratory failure and it is recommended that the ventilation mode decision should be made according to the clinical experience of the physician and the clinical characteristics of the patient.^1,2^ Pressure-regulated volume-control ventilation (PRVC) mode is defined as a dual-mode that combines the variable flow pattern of the pressure-control (PC) mode with the tidal volume guarantee properties of the volume-control mode (VC).^3^ The PRVC mode is defined as volume-targeted rather than volume-controlled because the peak pressure is readjusted by the device for each breath according to inspiratory tidal volume.^3^ The same amount of tidal volume can be achieved with lower peak pressures in PRVC than VC mode, due to the variable flow pattern. Pressure-controlled ventilation modes (assist/control or synchronized intermittent mandatory ventilation) are frequently preferred modes in pediatric mechanical ventilation practice.^4,5^ Anaesthesiologists’ choice of pressure-controlled modes due to the inability of delivering low tidal volumes with conventional (old) anaesthesia machines during pediatric anaesthesia procedures seems to be the origin of this practice.^6,7^ The studies conducted in pediatric intensive care units in Argentina and Italy in 2003 and 2007 showed that PRVC mode usage was not common in their daily pediatric intensive care practice.^8^ A recent study by Tekgüç et al.^4^ shows that pressure-controlled ventilation modes have been used more frequently in the weaning period than PRVC (almost 5%) in daily pediatric critical care practice in Turkey. The objective of this study is to present our clinical experience of using PRVC and PC ventilation modes in children. 

## Methods

**Figure 1.**
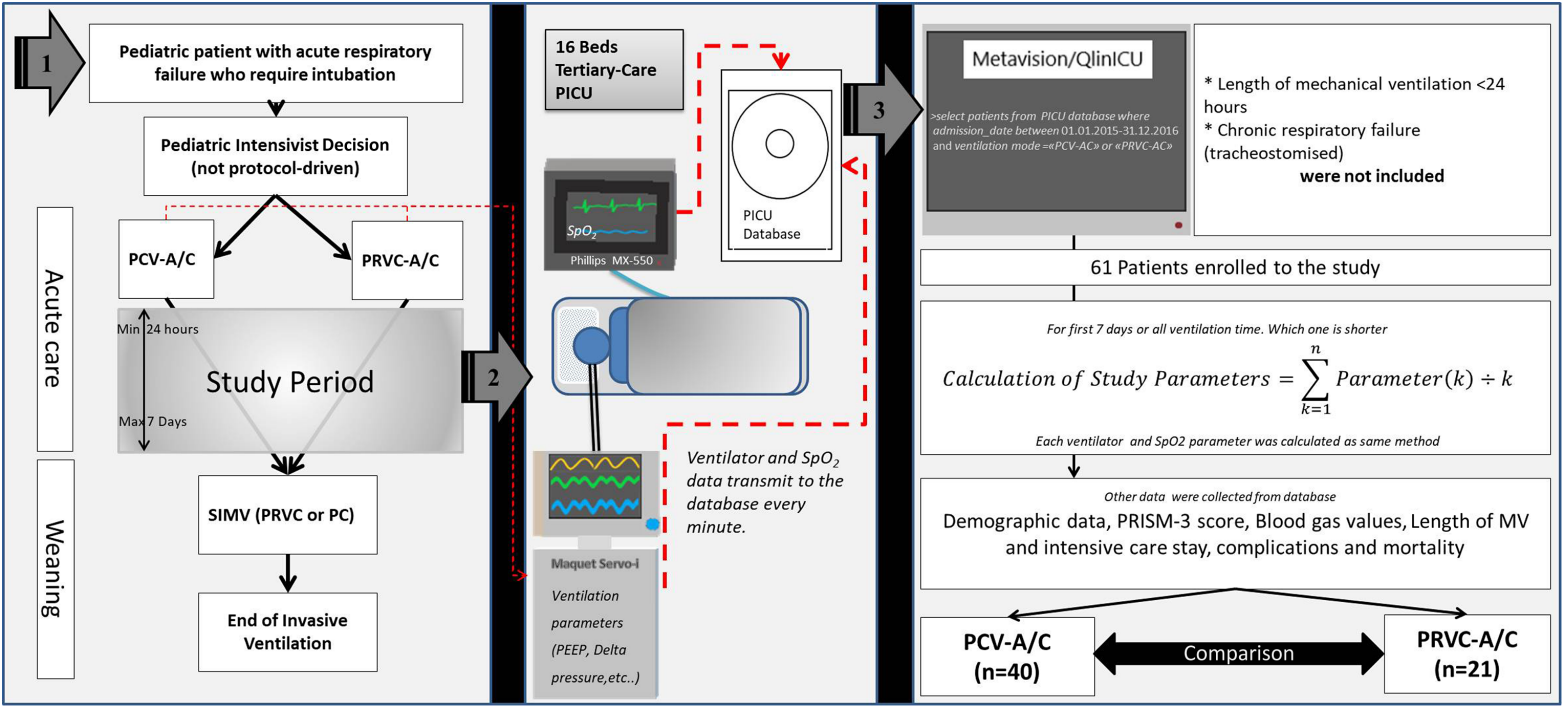
Graphical presentation of study method.

This study was reviewed and approved by the local ethical committee of Bakırköy Sadi Konuk Training and Research Hospital (no: 2019-19-07). Patients intubated due to acute respiratory failure and ventilated with pressure control–assist control or pressure-regulated volume control–assist control ventilation modes (Table 1) were retrospectively collected from PICU Metavision (IMDsoft, Israel) database of 1 year period (Figure 1). Patients with ventilation duration shorter than 24 hours and patients with chronic respiratory failure (tracheostomized and ventilator-dependent) were not included in the study. Both PC and PRVC (both assist-control and synchronized intermittent mandatory ventilation) ventilation modes have been routinely used for critically ill children in our PICU. The ventilation mode selection (PC or PRVC and assist-control or synchronized intermittent mandatory ventilation) has not been protocol-driven and it is based on the clinical findings of the patient and the decision of the pediatric critical care specialist. All adjusted, measured, and actual ventilator data transmit to the database on a 1-minute basis. Ventilation parameters (actual or measured parameters) from the mechanical ventilator (Maquet servo I, Sweden) and peripherally oxygen saturation from the bedside monitor (Phillips MX-550) were collected from the Metavision database for the first 7 days during the period of intubation follow up or all ventilation process if shorter than 7 days. During synchronized intermittent mandatory ventilation, data were not collected which was the weaning period in our clinical practice. All data were saved in the database on a 1-minute basis so all ventilator and SpO_2_ data could be collected as mean values for each patient. Plateau pressure and static compliance data could not be collected because the plateau pressure can only be measured in PC and PRVC modes by inspiratory hold maneuver and plateau pressure measurement was not our daily clinical routine for all ventilated patients in the study period. Blood samples were taken from the central venous line for blood gas analysis. The nadir pH (and accompanying HCO_3_ and base excess) and the peak pCO_2_ values during the follow-up period were collected for blood gas parameters. Hypercapnia was defined as pCO_2_>50 mm Hg. Hypoxemia was defined as oxygen saturation index ([mean airway pressure × FiO_2_]/sPO_2_) ≥ 5 for patients with sPO_2_ ≤ 97.^9^

### Statistical Analysis

Categorical variables were expressed as n (%) and continuous variables as median and interquartile range (25p-75p) or mean (standard deviation) according to normality. Kolmogorov-Smirnov test was used for the normality test. Comparisons of categorical variables between 2 groups were performed with chi-square or Fisher’s exact test which one is suitable. Comparisons of continuous variables between 2 groups were performed with Mann–Whitney *U* test for non-parametric variables and *t*-test for parametric (normally distributed) variables. Simple logistic regression analysis was performed for dependent variable mortality and results were expressed as odds ratio (OR), 95% CI for OR, and *P*-value. A *P*-value <.05 was considered statistically significant for all tests. The Statistical Package for Social Sciences (SPSS) version 23.0 software (IBM Corp.; Armonk, NY, USA) and Medcalc 14.8.1 for Windows were used for statistical calculations.

## Results

Sixty-one (42 boys and 19 girls) patients with a median age of 12 (4.8-36.4) months were included in the study (Table 2). Forty-one (67.2%) patients were aged below or equal to 24 months, 12 (19.7%) were aged 24-72 months, and 8 (13.1%) were aged above 72 months. Forty-eight (78.7%) patients had lower respiratory tract infections, 7 (11.5%) had central nervous system infections, 3 (4.9%) had heart failure, and 3 (4.9%) had traumatic injuries. Twenty-eight (45.9%) patients were diagnosed with hypoxemic respiratory failure and 44 (72.1%) were diagnosed with hypercapnic respiratory failure. The median pediatric risk of mortality score (PRISM-3) was 20 (27-24). Forty patients (65.6%) were ventilated in PCV and 21 (34.4%) in PRVC ventilation mode (Table 3). The length of mechanical ventilation was 71 (46-138) hours and the length of intensive care stay was 26 (14-42) days. Nineteen (31.1%) patients did not survive to discharge.

Single variable logistic regression analysis results for dependent variable mortality were as follows: hypoxemic respiratory failure, OR: 3.9 (95% CI 1.2-12.3, *P* = .02), pH (nadir), OR: 0.004 (95% CI: 0.000-0.275, *P* = .01), and base excess, OR: 0.88 (95% CI 0.79-0.98, *P* = .02). Used modes, neither PRVC nor PCV were not significant in logistic regression for mortality. 

## Discussion

Mean airway pressure and FiO_2_ are known as major determinants of oxygenation in mechanical ventilation. The best definition for mean airway pressure is the area under the curve of the pressure–time curve.^10^ Unlike VC mode, a constant pressure–time curve provides a constant mean airway pressure in pressure-controlled ventilation. Although this supports the superiority of the PC mode in hypoxemic patients, studies show no significant differences between PC and VC modes in hypoxemic respiratory failure.^11^ Pressure control mode was preferred more in hypoxemic patients rather than PRVC in our study and this is the reason for the differences (positive end-expiratory pressure, peak pressure, frequency, and FiO_2_) between the 2 modes. There were no significant differences between PC and PRVC mode ventilated patients in terms of length of mechanical ventilation, mechanical ventilation-associated complications, length of PICU stay, and intensive care mortality. This suggests that PRVC is as safe as PCV in children.

It is not expected that mortality can be explained by a single factor in the intensive care unit.^12^ Many different variables may affect mortality at different rates in different disorders. Oxygen is an extremely important molecule for the vitality of all living cells in the human body. With a sufficient cardiac output, hypoxia can be avoided in hypoxemic respiratory failure.^13^ However, the heart itself needs a significant amount of oxygen for sufficient work and that worsens the situation in long-term hypoxemia and hypoxia becomes inevitable.^14^ In our study, it was seen that the mortality risk increases 3.9 times in the presence of hypoxemic respiratory failure. The results of our study showed that the base deficit is inversely related to mortality, as a result, metabolic acidosis is a higher risk for mortality than respiratory acidosis in pediatric respiratory failure. 

Pressure-controlled modes are frequently used in daily pediatric practice in Turkey. Pressure-regulated volume-control mode is a volume-targeted and pressure-controlled ventilation mode and delta pressure is adjusted automatically for each breath, unlike the conventional PC mode. High peak pressures appear to be the most important reason why VC mode is not commonly used in pediatrics. There are some differences between VC and PRVC mode settings. In VC mode, any 2 of the 3 parameters (inspiratory time, inspiratory flow rate, and tidal volume) must be set as mandatory, in PRVC mode, tidal volume and inspiratory time must be set as mandatory. Although the PRVC mode delivers the same tidal volume with a lower peak pressure than the VC mode, the mean airway pressures are the same in both modes.^15^ However, there is no evidence in the literature that the PRVC mode is superior to the VC mode.^16^

The retrospective design of this study is an important limitation. Ventilation mode selection of physicians was not protocol-driven or randomized and this may have biased the results. 

## Conclusion

Our study showed that, although the PC mode was preferred more frequently in hypoxemic respiratory failure, there were no significant differences between the 2 respiratory modes in terms of length of PICU stay, mechanical ventilation duration, and mortality. The PRVC mode seems to be a safe option for children for physicians who do not have enough experience in using PC mode. However, it should be kept in mind that the target tidal volume in PRVC mode is inspiratory tidal volume and adequate ventilation may not occur in cases with leakage around tube or pneumothorax. It seems that more studies are needed to understand the advantages and disadvantages of the PRVC mode over conventional VC mode.

## Figures and Tables

**Table 1.** Definition of Assist-Control Ventilation Modes Mentioned in the Text and the General Rules of Setting the Parameters in Our Daily Clinical Practice

	**PC**	**PRVC**
**Positive End-Expiratory Pressure (PEEP) 1**	5 cmH_2_O	5 cmH_2_O
**Peak pressure 2**	15 cmH_2_O	N/A
**Tidal volume 3**	N/A	4-6 to 12 mL kg-1
**Target minute ventilation 4**	150-250 mL kg-1 min-1	150-250 mL kg-1 min-1
**Inspiratory time 5**	0.6-1 seconds	0.6-1 seconds
**Frequency 6**	15-30/min	15-30/min
**FiO 2 7**	As low as possible	As low as possible
^1^Peak end-expiratory pressure. High PEEP is used in hypoxemic respiratory failure. Low PEEP may be used in obstructive respiratory diseases according to auto-PEEP level. ^2^PEEP+Delta pressure. It should be an increase or decrease according to target tidal volume. ^3^Tidal volume should be 4-6 mL/kg with high frequency in restrictive disease or as high as 12 mL kg-1 with low frequency in obstructive disease. ^4^Age-dependent. High in infants and low in adolescents. ^5^Inspiratory time is dependent on age, type of lung disease, and respiratory frequency. It may be higher in adolescents. ^6^Age- and disease-dependent. It should be high with low tidal volumes in hypoxemic diseases and it should be low with high tidal volume in obstructive diseases. ^7^ FiO_2_ goal is ≤60%. PC, pressure control; PRVC, pressure-regulated volume control; N/A, none-applicable. **Assist-control (A/C) ventilation technic:** All breaths triggered by time (no spontaneous breathing) or flow/pressure trigger (spontaneous breathing) and equally (fully) supported with pressure control (PCV-A/C) or pressure-regulated volume control (PRVC-A/C) or volume control (VC-A/C). **A/C—pressure-control mode (PC):** Breaths are triggered by time or flow or pressure trigger and peak inspiratory pressure is adjustable with a characteristic square-shaped (variable flow) pressure–time curve. **A/C—volume-control mode (VC):** Breaths are triggered by time or flow or pressure trigger and tidal volume is adjustable with a characteristic triangle-shaped (constant flow) pressure–time curve. **A/C—pressure-regulated volume control mode (PRVC):** Breaths are triggered by time or flow or pressure trigger and peak inspiratory pressure is determined by the device according to adjusted tidal volume with a characteristic square-shaped (variable flow) pressure–time curve.		

**Table 2.** Demographic and Blood Gas Data of PC and PRVC Groups

	PC, n = 40	PRVC, n = 21	*P*
Age (months), median (IQR)	11.5 (4.9-41.03)	12.03 (2.1-28.9)	.8
Gender (male/female)	28/12	14/7	.78
PRISM^*^-3 24 score	22 (19-24)	19 (15-20)	.07
**Hypoxemic respiratory failure,** n (%)	**22 (55%)**	**6 (28.6%)**	**.04**
Hypercapnic respiratory failure, n (%)	29 (72.5%)	15 (71.4%)	.9
pH (nadir), median (IQR)	7.19 (7.06-7.28)	7.21 (7.03-7.29)	.9
Peak pCO_2_ (mmHg), median (IQR)	76.8 (65.2-96.8)	79 (58.3-102.6)	.9
HCO_3_ (mEq L-1), median (IQR)	28.8 (24,4-31,9)	30.3 (25.4-32.5)	.6
Base excess (mEq L-1), median (IQR)	3.89 (0.3-7.5)	5.76 (1.3-8.2)	.6
Peripherally oxygen saturation (%), median (IQR)	96.5 (95-97.3)	97 (96-97.5)	.17
Length of mechanical ventilation (hours) median, (IQR)	67 (39.6-137.5)	81.8 (62.3-156.6)	.1
Length of PICU stay (days) median, (IQR)	21 (14-42)	30 (16-42)	.43
Non-survivors, n (%)	15 (37.5%)	4 (19%)	.13

^*^Pediatric risk of mortality score.

PC, pressure control; PRVC, pressure-regulated volume control; IQR, interquartile range.

**Table 3.** Comparison of Ventilation Parameters of PC and PRVC Modes

		PC, n = 40	PRVC, n = 21	*P*
**Positive end-expiratory pressure (**cmH_2_O**)**, median (IQR)		**5.4 (4.2-6.3)**	**4.05 (3.68-4.41)**	<.001
Delta pressure (cmH_2_O), mean (SD)		15 (3.48)	13.5 (3.7)	.6
**Peak pressure (cmH 2 O), mean (SD)**		**20.8 (4.3)**	**17.7 (3.7)**	**.007**
Inspiratory time (seconds), median (IQR)		0.55 (0.46-0.72)	0.68 (0.53-0.83)	.054
Mean airway pressure (cmH_2_O), mean (SD)		10.3 (2.4)	8.3 (1.9)	.05
**Frequency (/min), mean (SD)**		**31.4 (7.5)**	**27.4 (7.1)**	**.02**
Inspiratory time/expiratory time (I/E ratio), median (IQR)		0.41 (0.33-0.48)	0.46 (0.36-0.51)	.42
Tidal volume expiratory (mL kg-1), median (IQR)		9.3 (7.4-11.4)	9.3 (8.8-10.9)	.96
Compliance (mL/cmH_2_O), median (IQR)		4.1 (2.4-5.6)	4.1 (3.1-7.03)	.36
**Fraction of inspiratory oxygen (%)**, median (IQR)		**61.1 (56.5-65.4)**	**55.3 (46.5-60.6)**	**.03**
Complications, n (%)	Pneumothorax	5 (12.5%)	1 (4.8%)	.7
	Pneumomediastinum	2 (5%)	1 (4.8%)	
Ventilator-associated pneumonia	4 (10%)	4 (19%)

PC, pressure control; PRVC, pressure-regulated volume control; IQR, interquartile range; SD, standard deviation.

## References

[1] Kneyber MCJ de Luca D Calderini E et al. Recommendations for mechanical ventilation of critically ill children from the Paediatric Mechanical Ventilation Consensus Conference (PEMVECC). Intensive Care Med. 2017;43(12):1764 1780. 10.1007/s00134-017-4920-z) 28936698 PMC5717127

[2] Duyndam A Ista E Houmes RJ van Driel B Reiss I Tibboel D . Invasive ventilation modes in children: a systematic review and meta-analysis. Crit Care. 2011;15(1):R24. 10.1186/cc9969) PMC3222058 21241490

[3] Branson RD Chatburn RL . Controversies in the critical care setting. Should adaptive pressure control modes be utilized for virtually all patients receiving mechanical ventilation? Respir Care. 2007;52(4):478 85. 17417981

[4] Tekgüç H Can FK Şik G et al. Daily practice of mechanical ventilation and weaning in Turkish PICUs: A Multicenter Prospective Survey. Pediatr Crit Care Med. 2020;21(5):e253 e258. 10.1097/PCC.0000000000002272) 32168304

[5] Farias JA Frutos F Esteban A et al. What is the daily practice of mechanical ventilation in pediatric intensive care units? A multicenter study. Intensive Care Med. 2004;30(5):918 925. 10.1007/s00134-004-2225-5) 15029473 PMC7095496

[6] Shahriari A Sheikh M . Is the pressure control mode for pediatric anesthesia machines really required? Anesthesiol Pain Med. 2016;6(2):e35350. 10.5812/aapm.35350) PMC4886522 27252907

[7] Feldman JM Optimal ventilation of the anesthetized pediatric patient. Anesth Analg. 2015;120(1):165 175. 10.1213/ANE.0000000000000472) 25625261

[8] Wolfler A Calderoni E Ottonello G et al. Daily practice of mechanical ventilation in Italian pediatric intensive care units: a prospective survey. Pediatr Crit Care Med. 2011;12(2):141 146. 10.1097/PCC.0b013e3181dbaeb3) 20351615

[9] Pediatric Acute Lung Injury Consensus Conference Group. Pediatric acute respiratory distress syndrome: consensus recommendations from the Pediatric Acute Lung Injury Consensus Conference. Pediatr Crit Care Med. 2015;16(5):428 439. 10.1097/PCC.0000000000000350) 25647235 PMC5253180

[10] Greenspan JS Shaffer TH Fox WW Spitzer AR . Assisted ventilation: physiologic implications and complications. In: Polin RA, Fox WW, Abman SH, eds. Fetal Neonatal Physiol. Elsevier; 2004:961 978.

[11] Chacko B Peter JV Tharyan P John G Jeyaseelan L . Pressure-controlled versus volume-controlled ventilation for acute respiratory failure due to acute lung injury (ALI) or acute respiratory distress syndrome (ARDS). Cochrane Database Syst Rev. 2015;1:CD008807. 10.1002/14651858.CD008807.pub2) 25586462 PMC6457606

[12] Orban JC Walrave Y Mongardon N et al. Causes and characteristics of death in intensive care units: A prospective multicenter study. Anesthesiology. 2017;126(5):882 889. 10.1097/ALN.0000000000001612) 28296682

[13] Sarkar M Niranjan N Banyal PK . Mechanisms of hypoxemia. Lung India. 2017;34(1):47 60. 10.4103/0970-2113.197116) 28144061 PMC5234199

[14] Fisher DJ Left ventricular oxygen consumption and function in hypoxemia in conscious lambs. Am J Physiol. 1983;244(5):H664 H671. 10.1152/ajpheart.1983.244.5.H664) 6846554

[15] Guldager H Nielsen SL Carl P Soerensen MB . A comparison of volume control and pressure regulated volume control ventilation in acute respiratory failure. Crit Care. 1997;1(2):75 77. 10.1186/cc107) 11056699 PMC28991

[16] Medina A Modesto-Alapont V del Villar Guerra P et al. Pressure-regulated volume control versus volume control ventilation in severely obstructed patients. Med Intensiva. 2016;40(4):250 252. 10.1016/j.medin.2015.07.010) 26391736

